# Use of the osmolal gap in diagnosing mixed physiology hyponatremia in a child with B‐cell acute lymphoblastic leukemia

**DOI:** 10.1002/ccr3.7428

**Published:** 2023-05-28

**Authors:** Jonathan M. Gabbay, Andrew E. Place, Maya Ilowite, Jia Zhu

**Affiliations:** ^1^ Department of Medicine Boston Children's Hospital Boston Massachusetts USA; ^2^ Harvard Medical School Boston Massachusetts USA; ^3^ Department of Pediatric Oncology Dana‐Farber/Boston Children's Cancer and Blood Disorders Center Boston Massachusetts USA; ^4^ Division of Endocrinology Boston Children's Hospital Boston Massachusetts USA

**Keywords:** hyponatremia, hypertriglyceridemia, precursor cell lymphoblastic leukemia‐lymphoma, inappropriate ADH syndrome

## Abstract

Hyponatremia is common among children undergoing treatment for hematologic malignancies and may be attributed to multiple underlying causes. In cases of hyponatremia due to mixed physiology, the osmolal gap, can identify pseudohyponatremia that may be masked by other causes.

## 1 INTRODUCTION

Moderate to severe hyponatremia, defined as a serum sodium concentration less than 130 mmol/L, is common in hospitalized children with acute lymphoblastic leukemia (ALL) and is associated with significant morbidity and mortality.^1^, ^2^ Causes of hyponatremia in children with ALL can often be attributed to treatment‐related complications, including dehydration and rehydration with intravenous fluids, severe infections, and syndrome of inappropriate secretion of antidiuretic hormone (SIADH).^2^, ^3^ Pseudohyponatremia can also occur due to hyperlipidemia, a potential side effect of ALL therapy, and is essential to assess, as clinical management would be tailored to the cause of pseudohyponatremia.^4^, ^5^, ^6^, ^7^ In cases of mixed physiology, the causes of hyponatremia may not be easily discerned from an initial clinical and laboratory evaluation.

Here, we present a case of a child with a new diagnosis of ALL who was found to have moderate hyponatremia attributed to multiple etiologies. Initial evaluation revealed hyponatremia and hypotonicity with concentrated urine, and an initial diagnosis of SIADH was made. At 24 h after presentation, the patient's serum samples were noted to be lipemic. The osmolal gap was elevated, consistent with the presence of unmeasured osmoles, and measurement of lipids confirmed hypertriglyceridemia. The whole blood sodium (direct ion‐selective electrode) was 132 mmol/L, which was discrepant from the plasma sodium of 122 mmol/L, confirming the additional diagnosis of pseudohyponatremia secondary to hypertriglyceridemia.

## 2 CASE HISTORY

A 12‐year‐old boy recently diagnosed B‐cell ALL presented to our hospital with hyperglycemia and hyponatremia. 4 weeks earlier, he was started induction chemotherapy (daily dexamethasone, vincristine, and PEG‐asparaginase) and was diagnosed with steroid‐induced hyperglycemia secondary to dexamethasone, which was treated with a subcutaneous insulin regimen as needed.

Prior to presentation, the patient and family report increased fatigue and polyuria, and nocturia two to three times per night. They endorsed baseline appetite and denied fevers, nausea, vomiting, diarrhea, constipation, or confusion. He reported intermittent hyperglycemia to 250 mg/dL in the post‐prandial period, and his insulin regimen had been adjusted accordingly at home.

On the day of presentation, he arrived to the Oncology clinic for routine labs and follow up (Table 1). He was found to have moderate hyponatremia (sodium 126 mmol/L, time [*t*] = 0 h, Table 1), hypochloremia (90 mmol/L), and hyperglycemia (267 mg/dL). Corrected sodium for hyperglycemia was 130 mmol/L.^8^ He was admitted to the hospital for further evaluation and management of presumed dehydration due to osmotic diuresis from hyperglycemia.

**TABLE 1:** Serum laboratory results for patient during hospital course given by time in hours. T is time given in hours from initial laboratory results. NM = not measured. NR = not reported. BUN = blood urea nitrogen. WB = whole blood.

	Initial (*t* = 0 h)	*t* = 8 h	*t* = 16 h	*t* = 24 h	*t* = 36 h
Sodium (mmol/L)	126	123	125	122	124
WB Sodium (mmol/L)	NM	NM	NM	132	133
Potassium (mmol/L)	3.89	3.75	3.55	NR	4.19
Chloride (mmol/L)	90	90	88	87	86
BUN (mg/dL)	20	16	22	19	17
Creatinine (mg/dL)	0.22	0.22	0.25	0.12	<0.10
Glucose (mg/dL)	267	149	133	147	192
Serum osmolality (mOsm/kg)	286	272	NM	NM	NM
Urine osmolality (mOsm/kg)	NM	566	778	812	906
Urine sodium (mmol/L)	NM	140	143	174	168
Osmolal gap	12	12	NM	NM	NM
(mOsm/kg)					

Upon arrival to the hospital, his heart rate was 116 beats per minute, blood pressure 127 over 85, respiratory rate 18 per minute, with oxygen saturation of 97% on air. His physical exam was notable for capillary refill of 2–3 s and a single area of oral mucositis that was healing. The remainder of his physical examination was unremarkable, including a normal neurological exam with intact mental status. Given his ongoing polyuria and tachycardia, he received normal saline bolus (20 cc/kg) for suspected dehydration. Repeat labs (*t* = 8 h, Table 1) showed a decreased serum sodium level of 123 mmol/L, serum glucose level of 149 mg/dL, and low serum osmolality of 272 mOsm/kg. His urine osmolality was concentrated at 566 mOsm/kg and urine sodium was elevated at 140 mmol/L. Endocrinology was consulted and his hypotonic hyponatremia with concentrated urine was thought to be at least partially attributed to SIADH. Of note, his serum osmolality was thought to be not as severely affected as the serum sodium. For the presumed diagnosis of SIADH, he was fluid restricted to 1 L/m^2^/day and started on sodium chloride supplementation of 1 g twice daily. Given the severity of hyponatremia, hypertonic saline was considered. However, the patient remained asymptomatic with normal mental status, and thus, hypertonic saline was not administered while the diagnostic evaluation was ongoing. Over the following 24 h, the serum sodium did not normalize (Table 1).

Approximately 24 h after admission, the laboratory reported gross lipemia. Whole blood sodium levels were obtained and noted to be mildly low at 132 mmol/L (*t* = 24 h; Table 1). The discrepancy between whole blood and serum sodium level suggested the presence of unmeasured osmoles that can lead to an artificial dilution of serum sodium levels. The osmolal gap,^9^ the difference between measured serum osmolality (reported lab value) and calculated serum osmolality (2[Na] + BUN/2.8 + Glucose/18) was calculated and found to be elevated at 12 mOsm/kg at *t* = 0 and 8 h, confirming the presence of unmeasured osmoles. The patient did not have evidence of metabolic acidosis that would be concerning for a toxic ingestion as the cause of his elevated osmolal gap. The recent administration of PEG‐asparaginase and grossly lipemic blood samples raised the concern for hypertriglyceridemia, a known side effect of PEG‐asparaginase therapy.^4^, ^5^, ^6^, ^7^ Triglycerides were found to be significantly elevated at 4840 mg/dL (*t* = 36 h), and therapy was started with omega 3 fatty acid and fenofibrate with improvement of his triglycerides to 109 mg/dL approximately 2 weeks later.

At 36 h of hospitalization, his whole blood sodium was 133 mmol/L; when corrected for the glucose of 197 mg/dL, the whole blood sodium was normal at 135 mmol/L. Thus, the patient's initial diagnosis of SIADH was thought to be either resolved or no longer clinically significant, and chloride supplementation and fluid restriction were discontinued. Sodium levels were monitored with no reoccurrence of hyponatremia at 2 weeks after discharge. For the patient's history of steroid‐induced hyperglycemia, glucose levels were monitored closely during hospitalization, and the patient did not require insulin.

## 3 DISCUSSION

Hyponatremia is a common complication in children undergoing treatment for acute lymphoblastic leukemia and can often be attributed to multiple etiologies.^2^, ^3^ For example, mucositis can lead to decreased oral intake and in severe cases, hyponatremia from dehydration. Hyperglycemia secondary to glucocorticoid treatment can cause osmotic diuresis, resulting in hyponatremia from dehydration. Hyperglycemia and the resulting hyperosmolality can also cause dilutional hyponatremia from diffusion of water into the extracellular space.^1^, ^10^ Chemotherapy agents, such as vincristine, are known to cause SIADH.^11^, ^12^, ^13^, ^14^ Severe infection can also lead to hyponatremia in critically ill patients.^1^ Finally, PEG‐asparaginase can cause hypertriglyceridemia, and, in severe cases, result in pseudohyponatremia.^4^, ^5^, ^6^, ^7^ These treatment‐related causes of hyponatremia can occur simultaneously, which can challenging to discern in the initial laboratory evaluation.^2^, ^3^ In our case of mixed physiology hyponatremia in a child undergoing induction therapy for ALL, we show how use of the osmolal gap can be used to identify pseudohyponatremia that may be initially masked by other causes.

In our patient, his initial hyponatremia and hypochloremia was thought to be at least partly due to dehydration from ongoing osmotic diuresis due to hyperglycemia based on the presenting history and exam. However, intravenous fluid administration with 20 mL/kg of isotonic fluids led to a further decrease in the patient's sodium levels from 126 mmol/L to 123 mmol/L. Timed serum and urinary studies showed a serum sodium of 123 mmol/L, low serum osmolality of 272 mOsm/kg, concentrated urine (566 mOsm/kg) and elevated urinary sodium (140 mmol/L), suggestive of SIADH physiology rather than dehydration.

In pediatric patients undergoing induction therapy, SIADH is a well‐known adverse effect of vincristine, which the patient recently received.^11^, ^12^, ^13^, ^14^ Thus, initial treatment with fluid restriction and sodium supplementation was implemented but did not improve the serum sodium after 24 h, which raised the concern that SIADH physiology was at least not the primary cause of hyponatremia. On review of his prior laboratory evaluation, the degree of hypotonicity was noted to be relatively mild (serum osmolality = 272 mOsm/kg) for the severity of the hyponatremia at the time (122 mmol/L). This discrepancy was confirmed by an elevated osmolal gap (>10 mOsm/kg), which was able to indicate the presence of unmeasured osmols, such as excess lipids, 24 h before the serum sample was noted to be grossly lipemic.^9^

Pseudohyponatremia is a spurious electrolyte abnormality due to artifactually reduced serum sodium in the setting of unmeasured osmoles, most commonly hyperlipidemia or hyperproteinemia.^15^, ^16^, ^17^, ^18^, ^19^, ^20^ Pseudohyponatremia due to hypertriglyceridemia has been described in several reports in children with hematologic malignancies following treatment with PEG‐asparaginase.^4^, ^5^, ^6^, ^7^ Serum sodium is commonly measured by indirect ion‐selective electrodes, which estimate the sodium content per total volume of plasma.^15^, ^16^, ^17^, ^18^ In the absence of unmeasured osmoles, the water phase containing sodium, composes approximately 92% of plasma, and indirect ion‐selective electrode methods calculate the serum sodium under this assumption. When hyperlipidemia is present, the water phase of plasma is relatively reduced, which leads to an artificial dilution of the sodium level in the total plasma and falsely low sodium levels when using indirect ion‐selective electrode methods. The effective sodium level in the water phase of plasma is normal.^18^, ^19^, ^20^ Serum osmolality is not affected by this measurement artifact and therefore, isolated pseudohyponatremia is characterized by normal serum osmolality. With direct ion‐selective electrodes, such as those used for whole blood gas, the sodium concentration is measured within the water phase of plasma only, which is not affected by hyperlipidemia.^18^, ^19^, ^20^

On initial evaluation, our patient had evidence of hypotonic hyponatremia. Although hypotonicity is not characteristic of pseudohyponatremia, pseudohyponatremia cannot be ruled out if there are other potential causes of hyponatremia present, such as SIADH. Furthermore, the presence of unmeasured osmoles was suggested by the relatively mild hypotonicity for the severity of the hyponatremia at initial evaluation. In our patient, calculation of the osmolal gap at the time of initial presentation could have led to the diagnosis of pseudohyponatremia 24 h before the serum was reported to be lipemic. Thus, diagnostic evaluation for hyponatremia using paired serum and urine studies and calculation of the osmolal gap is important in identifying all possible etiologies of hyponatremia in this complex patient population.

## AUTHOR CONTRIBUTIONS

**Jonathan M Gabbay:** Conceptualization; writing – original draft; writing – review and editing. **Andrew E Place:** Conceptualization; writing – review and editing. **Maya Ilowite:** Conceptualization; writing – review and editing. **Jia Zhu:** Conceptualization; funding acquisition; supervision; writing – review and editing.

## FUNDING INFORMATION

J.Z. was supported by grant 1F32HD103317 from the National Institute of Child Health and Human Development. The authors have no financial relationships to declare.

## CONFLICT OF INTEREST STATEMENT

The authors have no conflicts of interest to declare.

## CONSENT

Written informed consent was obtained from the patient's legal guardian for the publication of this case report.

## Data Availability

Data sharing not applicable to this article as no datasets were generated or analyzed during this study.

## References

[1] Wattad A , Chiang ML , Hill LL . Hyponatremia in hospitalized children. Clin Pediatr (Phila). 1992;31:153‐157. 1547587 10.1177/000992289203100305

[2] Janczar S , Zalewska‐Szewczyk B , Mlynarski W . Severe hyponatremia in a single‐center series of 84 homogenously treated children with acute lymphoblastic leukemia. J Pediatr Hematol Oncol. 2017;39(2):e54‐e58. 28060134 10.1097/MPH.0000000000000758

[3] Salvador C , Salvador R , Willeit P , et al. Hyponatremia during induction therapy in distinct pediatric oncological cohorts: a retrospective study. Front Oncol. 2021;11:708875. 34778028 10.3389/fonc.2021.708875 PMC8586428

[4] Hinson A , Newbern D , Linardic CM . Asparaginase‐induced hypertriglyceridemia presenting as Pseudohyponatremia during leukemia treatment. Case Rep Pediatr. 2014;2014:635740. 25405049 10.1155/2014/635740 PMC4227320

[5] Parsons SK , Skapek SX , Neufeld EJ , et al. Asparaginase‐associated lipid abnormalities in children with acute lymphoblastic leukemia. Blood. 1997;89(6):1886‐1895. 9058708

[6] Ghersin Z , Fernandes ND , Winkler A , Yager P . Pseudohyperkalemia and Pseudohyponatremia in two children with T‐cell acute lymphoblastic leukemia. J Pediatr. 2021;232:294‐298. 33493492 10.1016/j.jpeds.2021.01.044

[7] Urakami C , Matsuno R , Omachi T , Yamazoe T , Kaneko K . Mind the gap in hyponatremia! J Pediatr Hematol Oncol. 2021;43(5):e742‐e743. 33235151 10.1097/MPH.0000000000002024

[8] Hillier TA , Abbott RD , Barrett EJ . Hyponatremia: evaluating the correction factor for hyperglycemia. Am J Med. 1999;106(4):399‐403. 10225241 10.1016/s0002-9343(99)00055-8

[9] Turchin A , Seifter JL , Seely EW . Clinical problem‐solving. Mind the gap. N Engl J Med. 2003;349(15):1465‐1469. 14534340 10.1056/NEJMcps031078

[10] Healy SJ , Nagaraja HN , Alwan D , Dungan KM . Prevalence, predictors, and outcomes of steroid‐induced hyperglycemia in hospitalized patients with hematologic malignancies. Endocrine. 2017;56:90‐97. 28058528 10.1007/s12020-016-1220-2

[11] Nagappa M , Bhat RR , Sudeep K , Mishra SK , Badhe AS , Hemavathi B . Vincristine‐induced acute life‐threatening hyponatremia resulting in seizure and coma. Indian J Crit Care Med. 2009;13(3):167‐168. 20040817 10.4103/0972-5229.58545 PMC2823101

[12] West ZE , Castellino SM , Monroe C , Thomas AS , McCracken C , Miller TP . Quantifying the difference in risk of adverse events by induction treatment regimen in pediatric acute lymphoblastic leukemia. Leuk Lymphoma. 2021;62(4):899‐908. 33258395 10.1080/10428194.2020.1852471 PMC8012228

[13] Ellison DH , Berl T . Clinical practice. The syndrome of inappropriate antidiuresis. N Engl J Med. 2007;356(20):2064‐2072. 17507705 10.1056/NEJMcp066837

[14] Esposito P , Piotti G , Bianzina S , Malul Y , Dal Canton A . The syndrome of inappropriate antidiuresis: pathophysiology, clinical management and new therapeutic options. Nephron Clin Pract. 2011;119(1):c62‐c73. 21677440 10.1159/000324653

[15] Liamis G , Liberopoulos E , Barkas F , Elisaf M . Spurious electrolyte disorders: a diagnostic challenge for clinicians. Am J Nephrol. 2013;38(1):50‐57. 23817179 10.1159/000351804

[16] Weisberg LS . Pseudohyponatremia: a reappraisal. Am J Med. 1989;86(3):315‐318. 2645773 10.1016/0002-9343(89)90302-1

[17] Albrink MJ , Hald PM , Man EB , Peters JP . The displacement of serum water by the lipids of hyperlipemic serum; a new method for the rapid determination of serum water. J Clin Invest. 1955;34(10):1483‐1488. 13263427 10.1172/JCI103199 PMC438723

[18] Langhoff E , Steiness I . Potentiometric analysis for sodium and potassium in biological fluids. Clin Chem. 1982;28(1):170‐172. 7055905

[19] Steffes MW , Freier EF . A simple and precise method of determining true sodium, potassium and chloride concentrations in hyperlipemia. J Lab Clin Med. 1976;88:683688. 965812

[20] Worth HG . A comparison of the measurement of sodium and potassium by flame photometry and ion‐selective electrode. Ann Clin Biochem. 1985;22(4):343‐350. 3898973 10.1177/000456328502200402

